# The impact of transitive annotation on the training of taxonomic classifiers

**DOI:** 10.3389/fmicb.2023.1240957

**Published:** 2024-01-03

**Authors:** Harihara Subrahmaniam Muralidharan, Noam Y. Fox, Mihai Pop

**Affiliations:** ^1^Department of Computer Science, University of Maryland, College Park, MD, United States; ^2^Center for Bioinformatics and Computational Biology (CBCB), University of Maryland, College Park, MD, United States

**Keywords:** transitive annotation, taxonomic classification, naïve Bayes classifier, RDP classifier, data poisoning, error percolation

## Abstract

**Introduction:**

A common task in the analysis of microbial communities involves assigning taxonomic labels to the sequences derived from organisms found in the communities. Frequently, such labels are assigned using machine learning algorithms that are trained to recognize individual taxonomic groups based on training data sets that comprise sequences with known taxonomic labels. Ideally, the training data should rely on labels that are experimentally verified—formal taxonomic labels require knowledge of physical and biochemical properties of organisms that cannot be directly inferred from sequence alone. However, the labels associated with sequences in biological databases are most commonly computational predictions which themselves may rely on computationally-generated data—a process commonly referred to as “transitive annotation.”

**Methods:**

In this manuscript we explore the implications of training a machine learning classifier (the Ribosomal Database Project’s Bayesian classifier in our case) on data that itself has been computationally generated. We generate new training examples based on 16S rRNA data from a metagenomic experiment, and evaluate the extent to which the taxonomic labels predicted by the classifier change after re-training.

**Results:**

We demonstrate that even a few computationally-generated training data points can significantly skew the output of the classifier to the point where entire regions of the taxonomic space can be disturbed.

**Discussion and conclusions:**

We conclude with a discussion of key factors that affect the resilience of classifiers to transitively-annotated training data, and propose best practices to avoid the artifacts described in our paper.

## 1. Introduction

Public databases are at the core of virtually all computational analyses of genomic data sets. The identity of organisms in a sample, the function of genes, and the phenotypic consequences of a genomic variant (among many other tasks) are all predicted by comparing newly-generated data to previously-annotated sequences in a reference database. The annotation of genomic features with taxonomic, functional, or phenotypic information is performed through a range of computational approaches, such as nearest-neighbor search-based techniques (where a sequence receives the label of the database sequence it most closely resembles) or supervised machine learning (where a model is trained using pre-annotated sequences for each label, and new sequences are annotated based on their statistical fit to the corresponding model).

Ideally, every label associated with an entry in a database would be the result of a careful experimental characterization of the corresponding organism, gene, or protein. For example, in microbial taxonomy, each label is defined based on morphological and phenotypic features of organisms (Sanford et al., 2021). However, the number of entries in biological sequence databases that are labeled on the basis of experimental evidence represents a small minority among all entries. As an example, Mahlich et al. (2018) describe that only 6,835 out of over 200,000 enzyme sequences from the SwissProt database are annotated on the basis of an experimental confirmation of enzyme function. Furthermore, in many cases, labels (in this case Enzyme Commission, or E.C., numbers) assigned to enzyme sequences did not have a single experimentally-verified sequence in the database.

The vast majority of labels associated with biological sequences are determined computationally from labels already present in databases, labels that themselves may have been inferred computationally. This situation is commonly referred to as “transitive annotation” since a particular label may be determined through a (potentially long) transitive chain of computational predictions. As in the case of the game “telephone,” the longer the inference chain, the more likely it is that errors are introduced. While the fact that transitive annotation introduces errors is well appreciated (Iliopoulos et al., 2003; Salzberg, 2007; Schnoes et al., 2009; Promponas et al., 2015), the impact of these errors on machine learning models is less well understood. In this manuscript, we focus on taxonomic annotation using a naïve Bayes classifier developed for the annotation of 16S rRNA gene sequences—the Ribosomal Database Project (RDP) classifier (Wang et al., 2007). We chose this data set and classifier since they are established resources in microbial ecology, and are extensively used by researchers around the world. However, our general methodology and conclusions apply more broadly to any sequence-based machine-learning classifier.

## 2. Background

The introduction of errors in biological databases due to transitive annotation has been recognized since the 1990s. Doerks et al., for example, state in 1998: *Database searches are used to transfer functional features from annotated proteins to the query sequences. With the increasing amount of data, more and more software robots perform this task. While robots are the only solution to cope with the flood of data, they are also dangerous because they can currently introduce and propagate mis-annotations* (Doerks et al., 1998). An attempt to model the process through which errors are introduced was made by Gilks et al. (2002) within the context of a simple annotation strategy that assigns a new sequence the label of the database sequence that is closest to it. Schnoes et al. (2009) highlight the significant error levels in protein databases, largely attributing these errors to the computational prediction of functional labels, contrasting this finding with the low error rates in manually curated databases. Promponas et al. (2015) explore several types of errors in protein functional annotations that are introduced by annotation approaches that go beyond simple sequence similarity. These are just a few examples that demonstrate the general awareness in the bioinformatics community of the fact that transitive annotation approaches lead to errors. Most of the research in this space, however, focuses on functional annotation prediction, and largely assumes simple annotation algorithms. To our knowledge, the impact of transitive annotation on taxonomic annotation performed with machine learning models has not yet been explored.

Here we focus specifically on the RDP classifier, a naïve Bayes classifier (Wickramasinghe and Kalutarage, 2021) for 16S rRNA gene sequences (Wang et al., 2007). During the training phase, the RDP classifier evaluates the frequency of 8-mers (substrings of length 8) extracted from the training sequences, estimating the conditional probability of observing each 8-mer in a given taxonomic group. During classification, the 8-mers in a query sequence are used to estimate the probability that the query sequence originates from a particular taxonomic group, and the sequence is classified into the taxonomic group with the highest probability of a match. Given the large imbalance between the total number of 8-mers (65,536) and the number of 8-mers in any given sequence (~1,600 in a full-length 16S rRNA gene sequence), 8-mer frequencies are expected to be low in any given sequence and taxonomic group, thus the addition of a single sequence may have a significant impact on the distribution of 8-mers within taxonomic groups, and therefore on the classifier’s output. We empirically explore this intuition in the remainder of the manuscript.

## 3. Methods

### 3.1. Experimental design

We set out to mimic a common situation in bioinformatics research. Scientists analyze some biological samples and generate sequences from the organisms present in those samples. These sequences are assigned taxonomic labels using a classifier, and are then deposited in a public database, annotated with the corresponding taxonomic labels. At a later point, scientists may use some of these sequences and their annotation as part of the training data for a new version of the classifier. To better understand how the impact of a particular sequence on the classifier output depends on the distance between this sequence and the decision boundary between adjacent (in feature space) taxonomic labels, we also generated artificial sequences that span the “space” between biological sequences that have divergent labels. The details are provided below. Note that in our experiment we do not assume any knowledge about the sequences that were used to train the classifier in the first place, rather we simply explore the effect of adding one or more new sequences to the training set.

**Figure 1:**
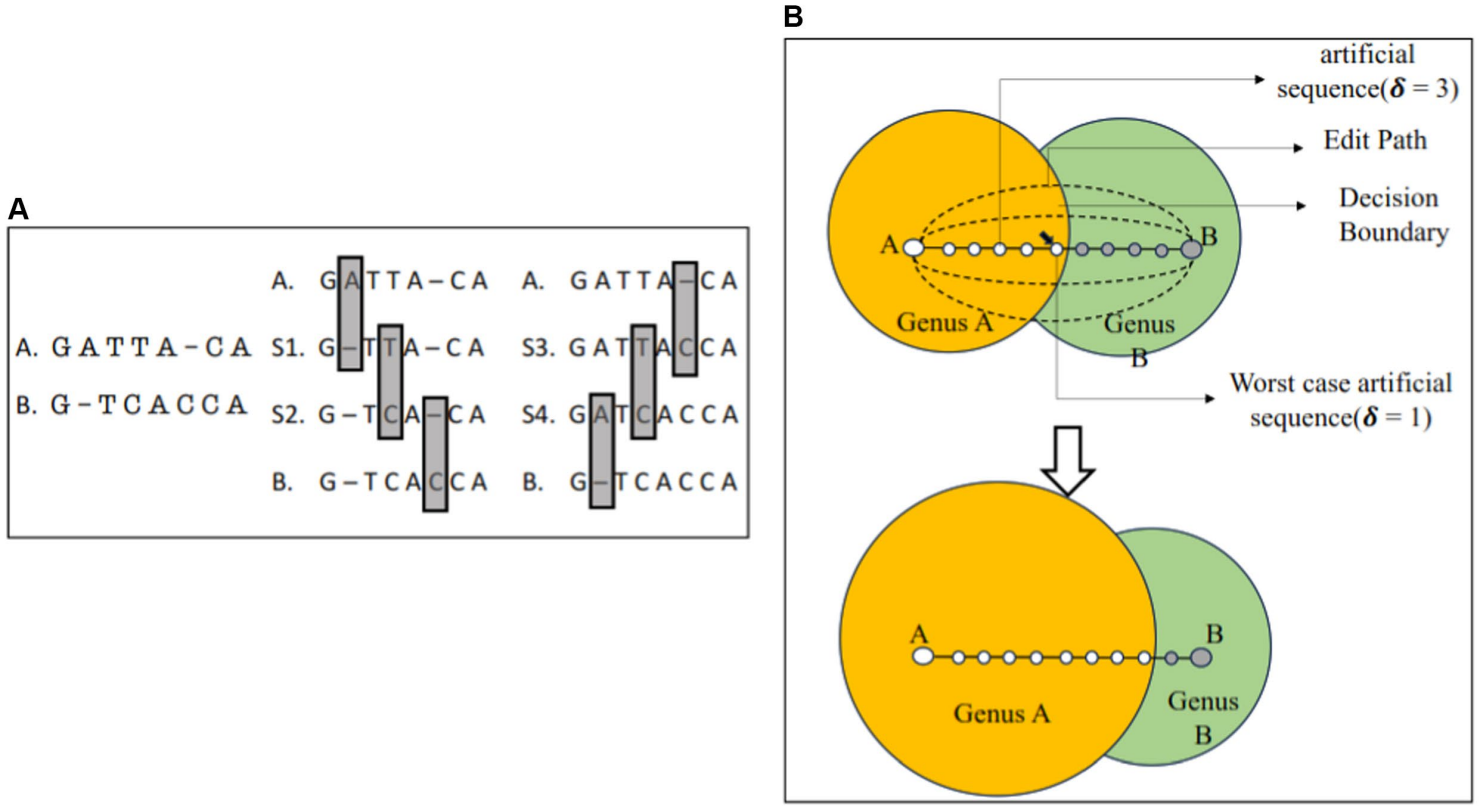
Overview of experimental setup. **(A)**: two sequences from the input **(A,B)** are aligned to each other, then multiple “edit paths” are computed between them by creating a set of sequences that differ between each other by exactly one edit, and that connect A to B. We show two such paths: A-S1-S2-B and A-S3-S4-B, which differ through the order in which the edits are made (highlighted by gray boxes). **(B)**: Modeling the impact of transitive annotation. Two sequences from the input that belong to distinct taxonomic genera A (empty circles) and B (gray circles) are aligned to each other and an edit path is computed (small circles). The transition in the label assigned to the intermediate sequences (change in shading) empirically identifies the location of the decision boundary that separates the two genera. A sequence close to the decision boundary (highlighted with a black arrow) is added to the training data for the classifier, resulting in a shift of the decision boundary, changing the label of the sequences previously belonging to genus B (reflected by a decrease in the size of the green region). The distance from a sequence to the decision boundary is denoted by δ.

We rely on a data set comprising 112,435 sequences of 16S rRNA gene operational taxonomic units (OTUs) generated in a study of childhood diarrhea in the developing world (Pop et al., 2014). These sequences were assigned taxonomic labels using the RDP classifier (version 2.5 obtained from https://github.com/rdpstaff/classifier), using the v18 version of its training dataset. All sequences were classified at the genus level, meaning the most specific classification we can obtain is a genus label. Within these data, we identified pairs of OTUs that are neighbors in sequence space yet were assigned divergent genus-level labels. Specifically, we identified 1,250 OTUs from 184 genera which formed 14,451 pairs of sequences that shared a family-level classification but diverged at the genus level, and that could be aligned to each other using BLAST (Altschul et al., 1990). We considered only alignments that could be obtained using BLAST with default parameters and where the alignment covered more than 85% of the shorter sequence. For each pair of aligned sequences, we then computed a set of 10 edit “paths”—chains of sequences that differ from the prior sequence by exactly one edit operation (substitution, insertion, or deletion of a nucleotide) and that together represent the chain of edits necessary to transform one sequence in the pair into the other (Figure 1A). We restricted our analyses to 10 distinct edit paths for the sake of computational tractability. Thus, each chain comprises the two (real) sequences in the pair, as well as a number of artificial sequences equal to the edit distance between the paired sequences. All artificial sequences were assigned taxonomic labels using the RDP classifier. Given that the original pair of sequences have divergent genus labels, this process allows us to empirically determine the distance (in number of edit operations) over which the classifier retains the same genus label as the endpoint of the edit path. In other words, we are effectively estimating the decision boundaries, within sequence space, of the labels assigned by the classifier. We then used this information to select sequences that are added to the training set for the classifier (Figure 1B), then explored the impact of the changes in training data on the genus-level boundaries identified within our data set. In order to emulate the transitive annotation process, each sequence added to the training data set was assigned the label provided by the classifier itself, i.e., the new training data simply reinforce the classifier’s initial prediction. We provide a flowchart and a detailed pseudocode used to identify candidate sequences for generating edit paths and the pseudocode to compute the edit paths in the Supplementary Figures S1–S3.

### 3.2. Experimental details

The training of the RDP classifier is orientation-specific, i.e., each sequence is processed in the “forward” orientation only (as defined according to the standard orientation of the *Escherichia coli* 16S rRNA gene). Since the experimental process used to generate the 16S rRNA gene sequences used in this study processed sequences in reverse, sequences were reverse complemented before being added to the training data set. Furthermore, the RDP classifier’s training data set comprises full-length 16S rRNA gene sequences, while the experimental data contain only a subset of this gene (between primers 338R and 27F, encompassing the hypervariable regions V1-V2) (Pop et al., 2014). When augmenting the training data, we added these partial sequences rather than trying to generate full-length versions of the 16S rRNA gene sequence by splicing the artificially-generated fragment into the backbone of the 16S rRNA gene. We made this choice for simplicity, and because pilot experiments demonstrated that the results are not affected by the length of the artificial sequence added to the training data set (results not shown).

## 4. Results

### 4.1. Proof of concept

**Figure 2:**
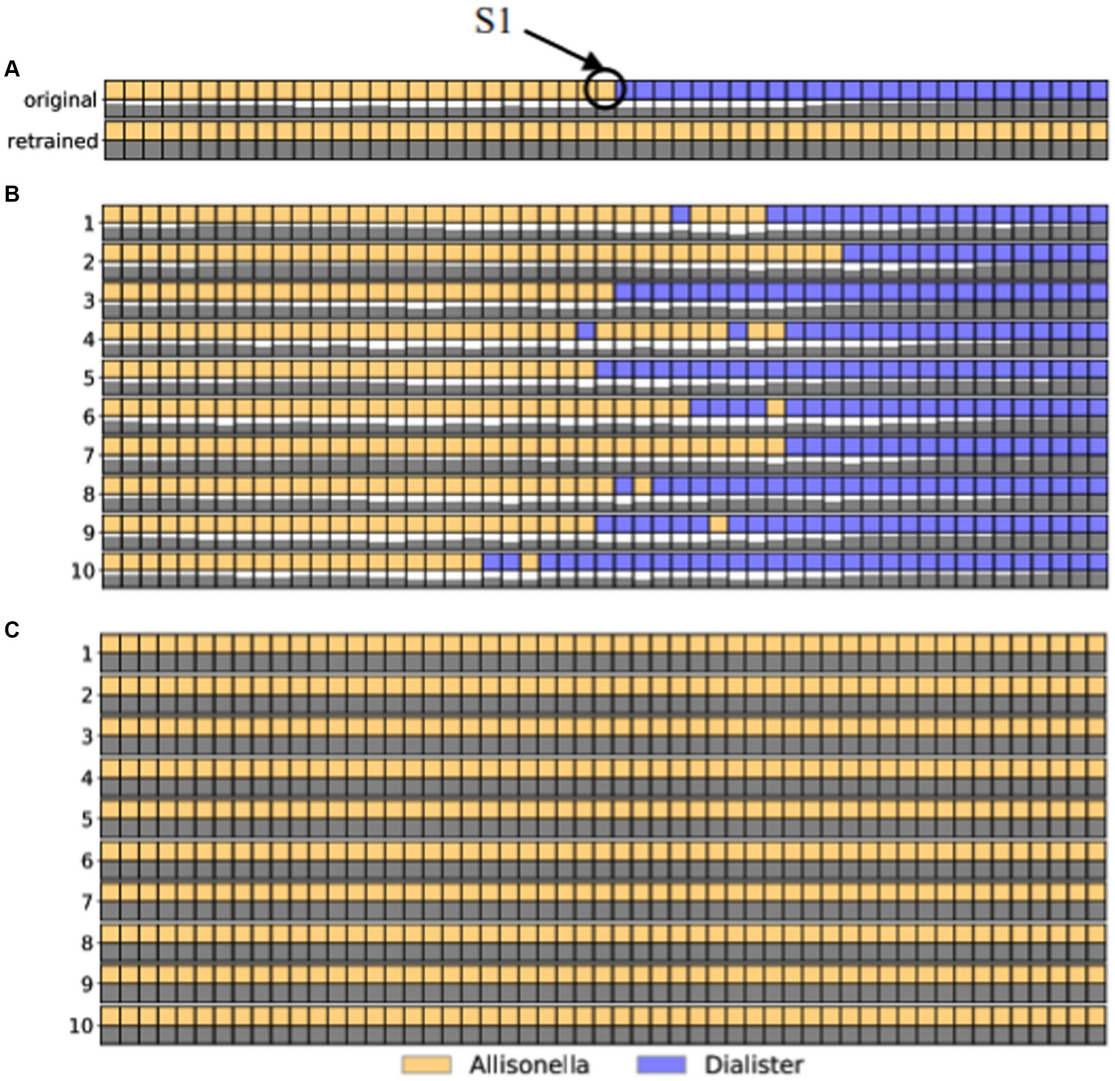
The effect of adding a single sequence (labeled S1 in panel **A**) to the training data set of the RDP classifier. Each row in the figure represents a set of edits between sequence 3928_5349(leftmost column) and sequence 3382_932. The colors correspond to the genus-level classification of each sequence, as shown in the legend. The shaded squares underneath each sequence represent the level of confidence in the classifier, from white (low confidence) to black (high confidence). **(A)** Path 3: The top two rows represent the original taxonomic labels and confidence values. The bottom two rows represent the taxonomic labels along this path obtained by adding sequence S1 to the training dataset. **(B)** The original taxonomic labels for 10 edit paths between the two sequences. **(C)** The taxonomic labels for all sequences along the 10 edit paths after the addition of sequence S1 to the training data set of the RDP classifier. As seen in all panels, the addition of this single sequence results in the change in labels of all sequences in the data, resulting in a significant increase in the number of sequences labeled *Allisonella*.

As a proof of concept, we highlight a pair of sequences from the childhood diarrhea data set, one classified in the genus *Dialister* (sequence 3382_932) and the other in the genus *Allisonella* (sequence 3928_5349). These sequences differ from each other by 53 edits. Thus, we generated 52 artificial sequences each differing from the next one by one nucleotide edits, covering the entire path between 3382_932 and 3928_5349. We computed ten different paths, each following a different order of edits, and classified each of the intermediate sequences at the genus level. The labels assigned to each intermediate sequence along the 10 paths are shown as different colors in Figure 2B. We focus on path 3 shown isolated in Figure 2A, that comprises sequences classified as *Allisneolla,* and *Dialister*, with the changes in color representing where the path crosses the decision boundaries for the RDP classifier.

From this path, we selected the sequence labeled S1 (annotated as *Allisonella*) and added this sequence to the training data set for the RDP classifier. After retraining the classifier, we re-annotated all the sequences, with the new labels shown in Figure 2C. Focusing just on path 3 (Figure 2A), we note that all the sequences previously labeled as *Dialister* are now labeled *Allisonella*. Furthermore, in the original data set, 1,811 OTU sequences were classified as *Dialister*. After re-training, 1,596 of these sequences were classified as *Allisonella*, demonstrating the significant impact of changing the decision boundary of the classifier through the addition of a single new sequence to the training data set.

### 4.2. Exploring the sensitivity of the RDP classifier

We define the sensitivity of the classifier as the number of sequences that change their label after the addition of one sequence to the training data set. As indicated above, the sequences added to the training data were either individual OTU sequences from the childhood diarrhea data set, or artificial sequences generated along an “edit path” between OTU sequences with divergent taxonomic labels. After retraining the classifier, we analyzed the number of sequences that changed taxonomic labels, as well as the taxonomic “distance” of the change, i.e., the level of the most recent common ancestor between the original label and the label assigned after re-training.

#### 4.2.1. Sensitivity depends on distance from decision boundary

**Figure 3:**
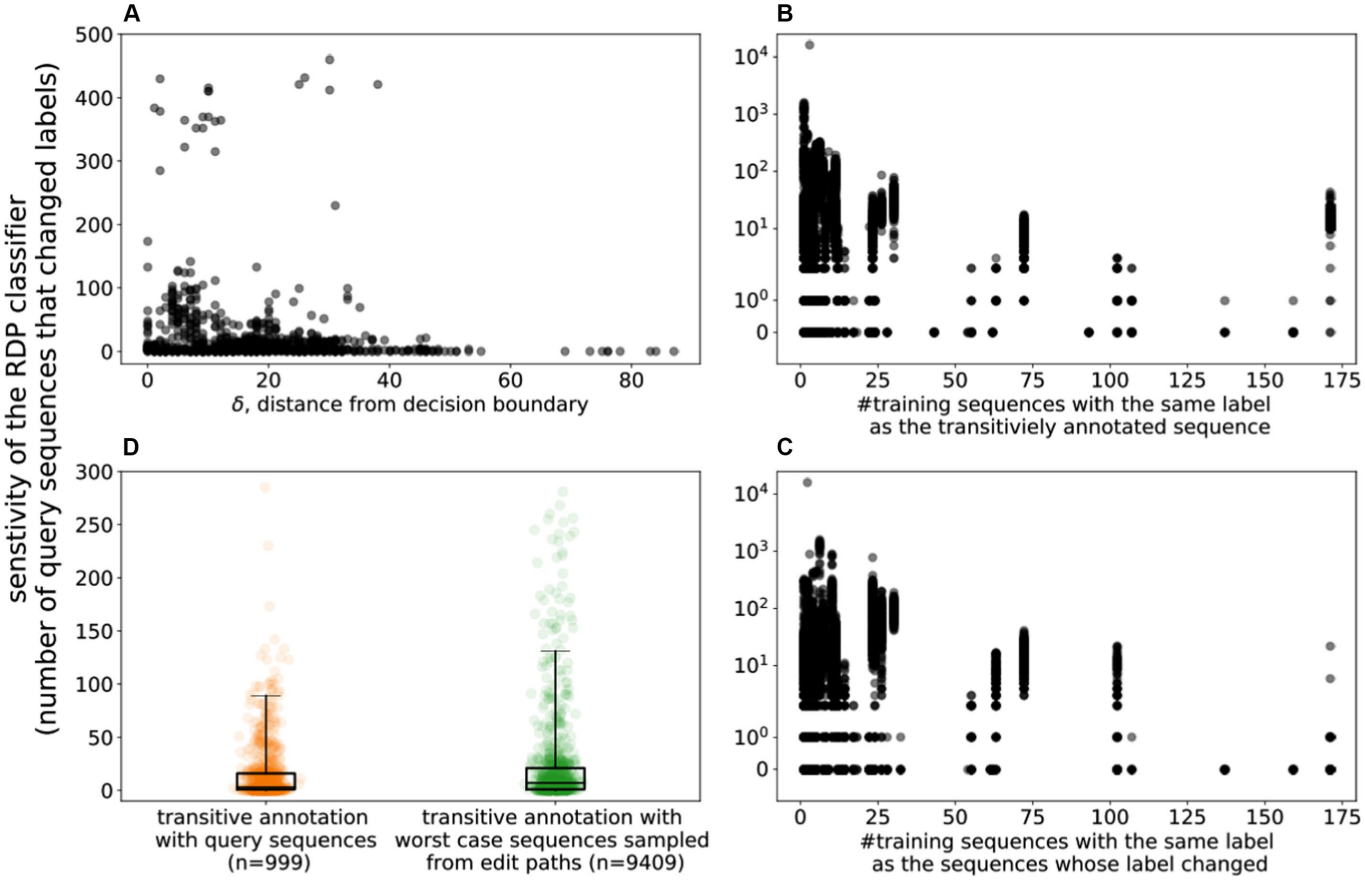
Sensitivity of the RDP Classifier. **(A)** The sensitivity of the classifier to the introduction of a single training sequence. X-axis: δ: the distance to the decision boundary (the number of edits needed for the taxonomic label of the sequence to change) Y-axis: sensitivity of the classifier (the number of sequences whose label changed upon adding a single training sequence) along the Y-Axis. **(B)** The effect of training class size on the sensitivity of the classifier. X-axis: the number of sequences that have the same label as the added training sequence; Y-axis: the sensitivity of the classifier. **(C)** X-axis: the number of sequences that have the same label as the sequence that changed labels upon adding one training sequence; Y-axis: the sensitivity of the classifier. **(D)** Comparing the impact of adding OTU (query) sequences to the training data with the impact of adversarially-created artificial sequences (The y-axis is trimmed to remove outliers).

We re-trained the RDP classifier after adding to the training data set, one by one, the 995 OTU sequences that had been paired with a sequence from a different genus. As described above, by traversing the edit path between the sequences, we are able to estimate the location of the classifier’s decision boundary between the two genera. Figure 3A highlights the relationship between the sensitivity of the classifier to the addition of a single training sequence and δ, the distance of this sequence from the decision boundary. As can be seen in the figure, the closer a training example is to the decision boundary, the more significant is its impact, in terms of the number of sequences that change labels after retraining the model (Pearson correlation between impact and distance to boundary is −0.13, *p* < 0.005).

#### 4.2.2. Sensitivity depends on support in training data set

The “strength” of the support in the training data, i.e., the number of training sequences that share a particular genus label, may impact the sensitivity of the classifier. Specifically, we addressed two questions: (i) does the impact of a newly added training sequence depend on the level of support of its label? (ii) does the level of support for a label influence its sensitivity to adversarial perturbations (likelihood the label is changed by a newly added training sequence)?

Figure 3B highlights the number of sequences that change labels as a new training sequence is added to the classifier, as a function of the level of support of the training sequence. As can be seen, the sensitivity of the classifier drops (Pearson correlation ρ −0.05, value of *p* <0.005) with the increase in the number of training sequences with the same label as the newly added example. In other words, adding new training examples to a label that is already well represented in the database has a lower impact than for less well represented labels.

Figure 3C highlights the number of sequences that change labels as a new training sequence is added, as a function of the level of support in the training data for the changed label. As can be seen, labels that are well supported in the training database are less sensitive to the addition of new training sequences for neighboring taxa.

#### 4.2.3. The inclusion of a single training sequence can affect distant taxa

To further substantiate the taxonomic magnitude of changes induced by the addition of a training sequence, we focused on a pair of taxonomic groups: *Streptococcus* and *Lactococcus*. We selected 238 pairs of sequences with divergent labels. From the edit path between pairs of such sequences, we selected the sequence labeled *Streptococcus* that was closest to the decision boundary between *Streptococcus* and *Lactococcus.* This sequence was then added to the training data set with the label *Streptococcus* (the same as the label assigned by the classifier). We note that the training database contained 171 and 24 sequences labeled as *Streptococcus* and *Lactococcus*, respectively. After re-training, we identified all the sequences that changed labels, and computed the most recent common ancestor between the original and the retrained label. From the 238 training sequences determined as described above, 233 (98%) caused changes that spanned microbial phyla. We observed 3,509 label changes after the inclusion of one or more training sequences, the majority (over 99.88%) of which changed to *Streptococcus*—an expected outcome given that the newly added training sequences had that taxonomic label. Note, however, that the data set comprised just 204 sequences labeled *Lactococcus*, thus most of the changes impacted genera that were not involved in the design of adversarial examples. Furthermore, several sequences changed labels to taxa other than *Streptococcus*. One sequence changed labels from *Lachnospiraceae incertae sedis* to *Clostridium* XVIa, one from *Clostridium* XVIa to *Lachnospiraceae incertae sedis*, one from *Ligilactobacillus* to *Pilibacter*, and interestingly, one sequence previously labeled as *Streptococcus* changed its label to *Haloplasma*.

More broadly, a total of 231,580 label changes occured after the addition to the training data set of one of 995 OTU sequences. A total of 2,481 (1.7%) such changes occur between different phyla, 1,048 (0.45%) between different classes, 57,267 (24.73%) between different orders, 9,434 (4.07%) between different families, and 161,350 (69.67%) between different genera. Some notable phylum level label changes include 272 sequences that changed from *Faecalibacterium* (phylum *Bacillota*) to *Succinivibrio* (phylum *Pseudomonadota*) and 109 sequences that changed from *Pseudescherichia* (phylum *Pseudomonadota*) to *Anoxybacillus* (phylum *Bacillota*).

#### 4.2.4. The sensitivity of the RDP classifier to “worst case” training examples

To explore the impact of training examples that are most likely to lead to prediction changes, we added to the training set sequences that were just one nucleotide away from the decision boundary between pairs of taxa. Specifically, we selected 995 sequences from the childhood diarrhea data set that were confidently labeled by the RDP classifier (classification accuracy of at least 0.8). Among these, we identified 9,358 pairs that had divergent genus-level labels, and that could be aligned to each other as described earlier. We then added to the training data set the sequence along the edit path that was closest to the decision boundary. We then compared the impact of adding this training example with that of adding its “parent” - the original OTU sequence from which the path originated. Figure 3D compares the distribution of changes to classifier predictions between the worst-case scenario (addition of sequence adjacent to decision boundary) and the “natural” experiment (addition of one of the OTU sequences to the training data). Selecting the sequences adjacent to the decision boundary results in a larger number of classification changes (KS-Statistic 0.12, *p*-value <0.005), with the median number of label changes increasing from 3 to 7. At the extremes, a sequence selected along the edit path between a *Barnesiella* and a *Coprobacter* OTU, caused 16,034 sequences to change labels, while an OTU sequence labeled *Massiliprevotella*, caused 11,594 sequences to change labels after its addition to the training data set.

#### 4.2.5. Transitive annotation impacts the labels of type strain sequences

Our analysis so far has focused on changes to the labels assigned to sequences derived from uncultured biological samples. To evaluate whether transitive annotation can impact the labels associated with sequences extracted from validated organisms, we analyzed a set of sequences obtained from type strain isolates. Specifically, downloaded type strain sequences from the SILVA database (specifying ‘[T] [T]’ in the query field) and re-classified them using the RDP classifier. We, then, focused on 107 sequences for which the SILVA and RDP classifications agreed. We then performed the same experiment as described in the experimental design section—we paired these sequences with sequences from different genera and created artificial training examples along the corresponding edit path. After retraining the classifier, 53 type strain sequences changed their label to a different genus. As a concrete example, sequence HQ286045 from the type strain *Streptomyces aidingensis* (Xia et al., 2013), changed its label to *Prauserella* after the introduction of a transitively annotated sequence labeled *Prauserella*, sequence obtained from the edit path between two type strain sequences AF466190 – *Prauserella* (Li et al., 2003) and AJ252832 - *Amycolatopsis* (Lee et al., 2000).

### 4.3. Studying the cumulative effect of training examples

The analysis so far focused on the effects of a single added training sequence. Here we explore the cumulative effect of adding multiple sequences to the training data of the classifier. Specifically, we focus on several “target” taxa and explore how much of these taxa can be obfuscated by the iterative addition of training examples from neighboring taxonomic groups.

**Figure 4:**
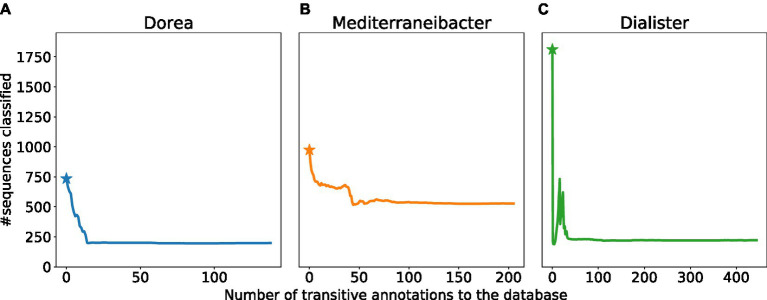
Cumulative effect of multiple training examples. Plots showing the number of sequences(Y-axis) classified as **(A)** Dorea **(B)**, Mediterraneibacter, **(C)** Dialister after the iterative addition to the training data set of new training examples (X-axis). The star indicates the number of sequences classified using the default database (prior to the addition of any new training sequences).

We focus on the genera *Dorea*, *Mediterraneibacter* and *Dialister*. For each of the three genera, we sampled multiple sequences from the edit paths connecting them to neighboring genera. For each such sequence, we evaluated the number of sequences from the target genus that changed their label. We then sorted these sequences in decreasing order of their impact, and added them, one by one, to the training data set, retaining the previously added sequences. The cumulative effect of these additions is shown in Figure 4. Specifically, we plot the number of sequences that are classified as *Dorea*, *Mediterraneibacter* and *Dialister* with the number of transitive annotations performed along the x-axis. We note that for all three taxa considered, the number of sequences whose labels change tends to saturate beyond a certain threshold. The point at which a particular genus label could not be degraded further by the addition of new training data varied across taxa: 199 for *Dorea*, 528 for *Mediterraneibacter*, and 222 for *Dialister*. We highlight the *Dorea* example in Supplementary Figure S4 where we focus on 6 *Dorea* sequences and their neighboring genera, together with the corresponding distances to the decision boundary. Also, the Supplementary Table S1 contains all sequences originally labeled as *Dorea* that eventually changed the label to a different genus. It is important to note, however, that our experiment does not represent a comprehensive “attack” on the specific labels, rather we sought to model a situation that can occur in practice as researchers populate the sequence databases with data from specific taxonomic groups.

## 5. Discussion

It is important to start by stating that we intentionally did not attempt to assess the accuracy of the predictions made by the RDP classifier and how this accuracy may be impacted by the addition of sequences to the training data. First and foremost, we did not want to delve into a possibly contentious debate about our definition of the correctness of taxonomic labels, particularly as the correctness of taxonomies itself is a debated topic (Hugenholtz et al., 2021). Second, we argue that precise measurements of accuracy are ultimately irrelevant to the main findings of our paper. We show that small changes to the data used to train the RDP classifier lead to significant changes in the classifier output, even though the new training data were labeled by the classifier itself. The fact that genus-level boundaries are sensitive to small perturbations in the training data implies errors are likely introduced as boundaries shift, an important observation even if the individual errors are not precisely identified.

As we have shown above, the impact on the classifier output of a single training sequence depends on three main factors: (i) the distance from the training sequence to a decision boundary (which we estimated empirically here); (ii) the number of training sequences that have the same label as the newly added sequence; and (iii) the number of training sequences that support the labels that may be impacted by retraining the model. These observations are not surprising given the way in which the naïve Bayes classifier used in our study operates. Since each taxonomic label is implicitly defined by the distribution of 8-mers within the training sequences with that label, factors (ii) and (iii) are consistent with the intuition that 8-mer distributions become more robust as more training examples are added. The first factor can be explained by the fact that the difference in 8-mer profiles of two sequences depends on the distance between them. Training examples that are nearby (in sequence space) sequences with divergent labels, share a large fraction of 8-mer profiles with them, thus having a stronger influence on classifier predictions when these 8-mers are added to the training data.

While we expected that the addition of a single training data point may have a local impact on the output of the classifier, we were surprised to see that some training examples led to the re-labeling of large numbers of sequences (exceeding 10,000 in one case), and that some of the changes traversed phylum boundaries. This observation implies caution is warranted when interpreting the output of automated annotation tools, particularly in the context of broad taxonomic surveys.

As we embarked on this project, we were concerned that an unlucky combination of transitively annotated sequences could lead a classifier to obscure one or more taxonomic groups. Our initial experiments demonstrate that erasing individual taxonomic groups is not trivially achieved, though we have not explored all possible “attacks.” Even so, the impact on the representation of individual taxonomic labels in the output of the classifier is quite significant, with more than half of the sequences originally classified in individual groups changing labels after adversarial training. This observation brings into question the accuracy of taxonomic profiling experiments, particularly when conducting meta-analyses that rely on different versions of classification tools.

Our findings underscore the importance of careful training of classifiers. We recommend here a few best practices in the context of taxonomic annotation, however readers are encouraged to also explore additional resources developed in different contexts (Wang et al., 2020; Artrith et al., 2021) As much as possible, the training data should be derived from experimental evidence, and computationally-derived labels should only be used when absolutely necessary (e.g., when the genomes analyzed are obtained from metagenomic samples rather than cultured specimens). Cross-validation and/or sensitivity analysis should be used to ensure that the classifier output is robust to small changes in the training data, and to identify the training examples that have a significant impact on the output so that these examples can be validated. Furthermore, both the classification software and the training database must be properly versioned and this information clearly documented in publications in order to enable meta-analyses. If the precise taxonomic label of sequences is important for a study (e.g., in the context of pathogen identification), or if precise abundance measurements need to be obtained, it is preferable to create specialized classifiers for the taxa of interest, which can be more effectively validated than general purpose multi-class classifiers.

We want to very clearly state that our study is just a start and we have only scratched the surface of the impact of transitive annotation on classifier output. We have limited our analyses to a random sample of sequences (and corresponding taxonomic labels) in order to limit the computational cost of our analyses, and only focused on one type of classification strategy—supervised learning using a naïve Bayes classifier. Additional research is needed to better understand the interplay between the accuracy of training data and classifier performance for a broader set of commonly-used classification algorithms used in genomics, including semi-supervised approaches (Triguero et al., 2015). It is also important to build bridges between the bioinformatics community and the broader research on data poisoning taking place in other fields of computing (Wang et al., 2022). Such cross-field interactions are particularly relevant due to recent advances in artificial intelligence. Impressive new technical capabilities made possible by these advances are coupled with new vulnerabilities that must be rapidly understood and overcome in order to allow the safe use of this technology in biomedical applications.

## Data availability statement

The datasets presented in this study can be found in online repositories. The names of the repository/repositories and accession number(s) can be found at: https://obj.umiacs.umd.edu/transitive-annotation/index.html.

## Author contributions

MP and HM designed the study and wrote the manuscript. MP, HM, and NF ran experiments and interpreted the data. All authors contributed to the article and approved the submitted version.
