# Supplementary material for: The impact of transitive annotation on the training of taxonomic classifiers
Source: Front Microbiol. 2024 Jan 3;14:1240957. doi: 10.3389/fmicb.2023.1240957 (PMC10792039; doi:10.3389/fmicb.2023.1240957)
Supplement: Supplementary file 1 [file Data_Sheet_1.PDF]

# 1 Supplementary methods and pseudocode

We describe an overview of the methods in this section. We present a simple workflow to perform transitive annotation in figure S1. We describe in detail, the pseudocode used to identify the candidates for generating edit paths and a pseudocode to generate the edit path in figures S2 and S3 respectively.

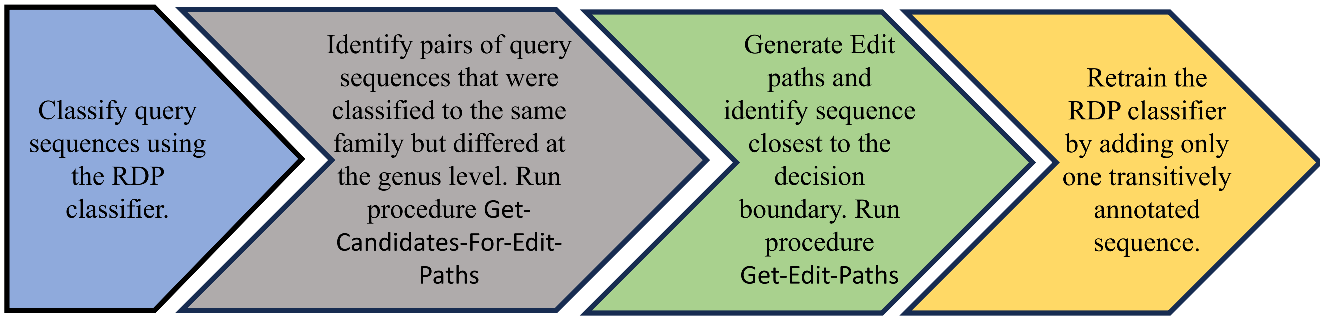


**Figure S1:** Flow chart depicting the overview of methods.

**Get-Candidates-For-Edit-Paths**

**Inputs**: List of Query Sequences, $Q$
Dictionary of Family labels for the query sequences, $\mathcal{F}$
Dictionary of Genus labels for the query sequences, $\mathcal{G}$
Dictionary of RDP confidence values for the query sequences at the genus level, $\mathcal{C}$
Maximum number of sequences to consider belonging to each family, NTAX = 10
RDP classifier confidence, $c = 0.80$
**Output**: Candidate sequence pairs for generating edit paths, $\mathcal{P}$

$\mathcal{D} = \{\}$ // Dictionary of sequences at different family labels
$\mathcal{P} = \{\}$
**for** $q \in \mathcal{Q}$
    **if** $\mathcal{C}[q] \geq c$
        $\mathcal{D}[\mathcal{F}[q]].insert(q)$
**for** $f \in \mathcal{D}$
    $S = \mathcal{D}[f]$
    **if** $|S| >$NTAX
        $S$ = Sample($S$, NTAX)
    **for** $i = 0$ To $|S| - 1$
        **for** $j = i + 1$ **to** $|S|$
            **if** $\mathcal{G}[S_i] \neq \mathcal{G}[S_j]$
                $\mathcal{P}[(\mathcal{G}[S_i], \mathcal{G}[S_j])] = \mathcal{P}[(\mathcal{G}[S_i], \mathcal{G}[S_j])] \cup (S_i, S_j)$
**return** $\mathcal{P}$

**Figure S2:** Pseudocode to identify candidates for generating edit-paths.

**Get-Edit-Paths**

```
Inputs: Sequence A
        Sequence B
        Number of edit paths, NPATHS = 10
        Coverage threshold, δ = 0.85
Output: List of artificial sequences, S
Aln =BLASTN(A, B)
if (Aln.align_length/Aln.glen < δ) OR (|Aln.qseq| ≠ |Aln.hseq|)
    return
q, h =Aln.qseq, Aln.hseq
mutations = [ ]
for i = 0 to |q|
    if q_i ≠ q_i
        mutations.insert((i, q_i))
mutations_order = [0, 1, ...., |mutations| − 1]
for i = 0 to NPATHS
    Shuffle(mutations_order)
    CurrSeq = q
    indels = 0
    for j = 0 to |mutation_order|
        pos, c = mutations[mutation_order[j]]
        if c ≠ '-'
            CurrSeq[pos-indels] = c
        else
            delete CurrSeq[pos-indels]
            indels = indels+1
        S.insert(CurrSeq)
return S
```

**Figure S3:** Pseudo-code to generate edit paths between sequences A and B.

## 2 Studying the effects of multiple transitive annotations on *Dorea.*

We describe the effects of multiple transitive annotations and exemplify it with specific examples. We provide more details on the specific experiment concerning *Dorea* in this supplementary section. we identified 6 sequences classified as *Dorea*, 3 sequences as *Mediterraneibacter*, 5 sequences classified as *Blautia*, 5 sequences classified as *Lachnospira*, 1 sequence originally classified as *Hungatella*, 7 sequences classified as *Agathobacter*, 2 sequences originally classified as *Anaerobutyricum*, 5 sequences classified as *Roseburia*, 2 sequences classified as *Anaerostipes*, and, 2 sequences classified as *Anaeritignum.* All these sequences generate a total of 138 edit paths. For each of the 6 *Dorea* sequences we show the average distance from the decision boundary from its neighboring taxa in figure S4. We observe a total of 547 sequences that were originally classified as *Dorea* changes labels. We summarize the sequences that change labels along with the genus it changes into, the minimum number of transitive annotations required to cause the label change and the taxa of the transitive annotation in the supplementary table[Dorea-Supplementary-Table.xlsx].

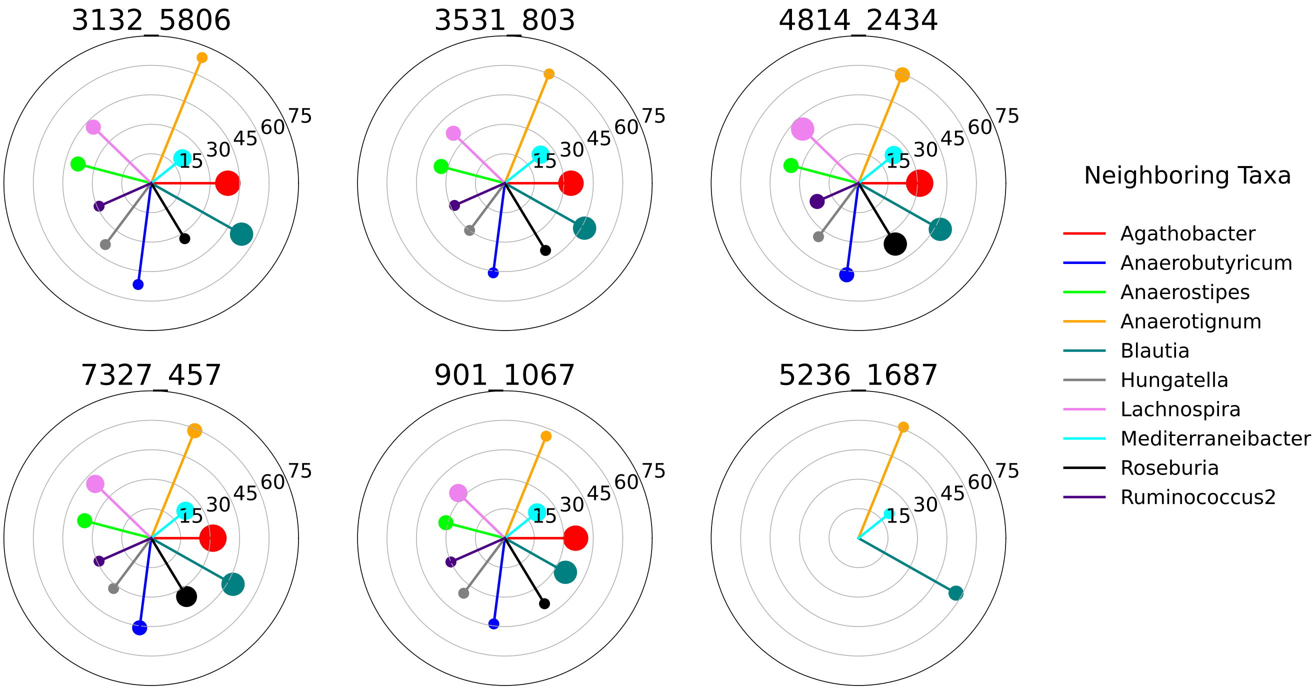


**Figure S4:** The distance from the decision boundary for each of the 6 *Dorea* sequences from the neighboring taxa. The radial lines represent the average distance of the Dorea sequence from the decision boundary of respective the neighboring taxa. The size of the dot is proportional to the number of artificial sequences with the corresponding taxa label.
